# The effect of sleep and shift work on the primary immune response to messenger RNA‐based COVID‐19 vaccination

**DOI:** 10.1111/jsr.14431

**Published:** 2024-12-10

**Authors:** Tamara M. J. Brouwers, Ümmü Gülsüm Çobanoğlu, Daryl Geers, Wim J. R. Rietdijk, Lennert Gommers, Susanne Bogers, Gert Jan Lammers, Gijsbertus T. J. van der Horst, Inês Chaves, Corine H. GeurtsvanKessel, Birgit C. P. Koch, Rory D. de Vries, Debbie van Baarle, Hugo M. van der Kuy, Heidi M. Lammers‐van der Holst

**Affiliations:** ^1^ Center for Infectious Disease Control National Institute for Public Health and the Environment Bilthoven The Netherlands; ^2^ Department of Medical Microbiology and Infection Prevention University Medical Center Groningen Groningen The Netherlands; ^3^ Department of Hospital Pharmacy Erasmus University Medical Center Rotterdam The Netherlands; ^4^ Department of Viroscience Erasmus University Medical Center Rotterdam The Netherlands; ^5^ Department of Neurology Leiden University Medical Center Leiden The Netherlands; ^6^ Sleep Wake Center Stichting Epilepsie Instellingen Nederland Heemstede The Netherlands; ^7^ Department of Molecular Genetics Erasmus University Medical Center Rotterdam The Netherlands; ^8^ Department of Public Health Erasmus University Medical Center Rotterdam The Netherlands

**Keywords:** shift work, COVID‐19 vaccination, night shift workers, primary immune response, sleep

## Abstract

Shift work can cause circadian misalignment, which often results in sleeping problems and has been associated with immune dysfunction. To better understand the impact of shift work on a primary immune response to vaccination, we compared severe acute respiratory syndrome coronavirus‐2 (SARS‐CoV‐2)‐specific humoral and cellular immune responses after one injection of the messenger RNA (mRNA)‐1273 vaccine between day workers (*n* = 24) and night shift workers (*n* = 21). In addition, duration and quality of sleep were assessed for a period of 7 days around the time of vaccination using actigraphy and daily sleep diaries, and their relationship with immunogenicity of mRNA‐1273 vaccination was studied. We found that median total sleep time on the 2 days immediately after vaccination, which coincided with the days that night shift workers worked night shifts, was significantly lower in night shift workers (342 and 318 min) than day workers (431 and 415 min) (both *p* < 0.001). There was no difference in sleep quality between day workers and night shift workers. Furthermore, no difference in the antibody response between the two groups was observed, yet night shift workers had a significantly higher virus‐specific T‐cell response than day workers 28 days after immunisation (*p* = 0.013). Multivariate regression analysis showed no association between sleep duration, sleep quality and SARS‐CoV‐2‐specific humoral or cellular immune responses. Collectively, these findings indicate that shift work‐induced sleep loss and night shift work have little to no effect on the primary immune response to mRNA‐based COVID‐19 vaccination.

## 1 INTRODUCTION

Humans have an internal circadian system that imposes near 24‐h rhythmicity on a vast number of physiological processes (Sukumaran et al., 2010). To keep pace with the exact 24‐h day–night cycle, the circadian system is reset daily by rhythmic environmental cues (known as ‘Zeitgebers’), such as light and food intake (Lee et al., 2021; Wang, Lutes, et al., 2022). Probably the most well‐known circadian‐controlled mechanism is the sleep–wake cycle (Beersma & Gordijn, 2007; Lee et al., 2021). Our ability to stay awake during the day and sleep at night is regulated by an interaction between the circadian timing system and a homeostatic sleep process (Fuller et al., 2006; Schwartz & Klerman, 2019). When the circadian phase and sleep homeostasis are aligned, sleep latency is short and there is consolidated sleep without significant wake. However, shift workers in hospital settings, police forces, and other night‐time professions have an irregular sleeping pattern because of continuously switching between night, evening, and day shifts. These atypical work schedules lead to misalignment between internal circadian time and the timing of a shift worker's sleep, resulting in shorter and less consolidated sleep, increased sleepiness, reduced performance during the shift work, and when severe, in shift work disorder (Akerstedt & Wright Jr., 2009; Kecklund & Axelsson, 2016). In line with this, shift work has been associated with overall disruption of homeostatic processes, immune dysfunction, and a wide range of diseases, such as cardiovascular diseases, metabolic syndromes and cancer, due to circadian misalignment (Baron & Reid, 2014; Kecklund & Axelsson, 2016; Schmitz et al., 2022).

There is increasing evidence that sleep can influence immune function (Besedovsky et al., 2019). Although findings are mixed, multiple studies have reported that sleep loss is associated with an enhanced pro‐inflammatory state (Besedovsky et al., 2019; Irwin et al., 2006; Irwin et al., 2023; Meier‐Ewert et al., 2004). Also shift work has been associated with changes in immune parameters. Loef, Nanlohy, et al. (2019) found that the mean number of monocytes in the blood was significantly higher in shift versus non‐shift workers. Moreover, in shift workers who recently worked night shifts, the level of lymphocytes, T cells and CD8^+^ T cells was significantly increased (Loef, Nanlohy, et al., 2019). Shift workers also have a higher incidence rate of severe respiratory infections and are thought to be more susceptible to acquiring a severe acute respiratory syndrome coronavirus‐2 (SARS‐CoV‐2) infection than non‐shift workers (Fatima et al., 2021; Loef, van Baarle, et al., 2019).

Moreover, both sleep and shift work seem to affect the immune response to vaccination. A recent meta‐analysis (Spiegel et al., 2023), which included four experimental studies (Benedict et al., 2012; Lange et al., 2003; Lange et al., 2011; Spiegel et al., 2002) and three prospective studies (Ayling et al., 2018; Prather et al., 2012; Prather et al., 2021), revealed that objective short sleep duration was associated with a decreased antibody response following influenza and hepatitis vaccination in adults aged 18–60 years. A significant association was also found between self‐reported short sleep and a reduced vaccine‐induced immune response, although this was only the case after exclusion of individuals aged ≥65 years and the effect was smaller compared to when sleep was measured objectively. Interestingly, Ruiz et al. (2020) showed that following immunisation with the meningococcal group C conjugate vaccine, night workers had a weaker humoral immune response than day workers (Ruiz et al., 2020; Schmitz et al., 2022). However, the difference in meningococcal serogroup C‐specific immunoglobulin G (IgG) levels between pre‐ and post‐vaccination timepoints might have been shaped by baseline antibody titres, which were significantly higher in night workers than day workers (Ruiz et al., 2020). Similar studies in the context of other vaccines, such as influenza vaccination, would also encounter the obstacle of pre‐existing immunity due to repeated previous exposure (Wild et al., 2021). Variability in baseline antibody titres can generally be addressed by having a sufficient sample size, which increases statistical power and allows for a more accurate assessment of immune responses. However, recruiting a large cohort can be challenging for vaccination studies, due to the relatively invasive procedures the participants must undergo. Research on the effects of circadian misalignment on the immune response to vaccination is scarce, but remains of great importance, especially because shift work is increasing in the current 24‐h society. The emergence of SARS‐CoV‐2 and the coronavirus disease 2019 (COVID‐19) pandemic provided the opportunity to investigate the effect of sleep and shift work on the immune response to a neo‐antigen.

The aim of this study was to compare the induction of SARS‐CoV‐2‐specific antibody levels and T‐cell responses following messenger RNA (mRNA)‐based COVID‐19 vaccination between day workers and night shift workers. In addition, we aimed to characterise the relationship between the duration and quality of sleep, and primary immunogenicity of mRNA‐based COVID‐19 vaccination in both day workers and night shift workers.

## 2 METHODS

### 2.1 Ethics statement

This prospective observational study (European Union Drug Regulating Authorities Clinical Trials Database [EudraCT] number 2021‐001395‐42) assessed the relationship between sleep, shift work and the primary immune response following mRNA‐based COVID‐19 vaccination in healthcare workers from the University Medical Center and police officers from the Dutch national police department in Rotterdam. This study was approved by the medical ethical committee of the Erasmus University Medical Center and the Central Committee on Research Involving Human Subjects (MEC‐2021‐0214). All participants provided written informed consent before the start of the study.

### 2.2 Participants

Before admission, people interested in participating were asked to complete an online screening survey to assess eligibility for the study. Volunteers were eligible for enrolment if they were aged between 18 and 50 years and worked ≥20 h/week (Lammers‐van der Holst et al., 2022). The inclusion criteria for night shift workers were: work schedules that included night shifts (defined as ≥6 h between 10:00 p.m. and 7:00 a.m.) or rotating shifts (morning, evening and night shifts), 6–12 h shift durations and ≥3 months history of working night or rotating shifts prior to the study. The inclusion criteria for day workers were: an average sleep duration of ≥7 h, regular bedtimes between 9:00 p.m. and 1:00 a.m. on workdays, regular wake times between 5:00 a.m. and 9:00 a.m., and no history of night shift work or rotating shifts within 6 months prior to the study. Volunteers were excluded if they previously had a severe reaction to a vaccine, had a SARS‐CoV‐2 infection within 3 months prior to the study, were pregnant or had a wish to become pregnant within 6 months, were breastfeeding, were at high risk of bleeding, were diagnosed with a sleep disorder, used medication known to affect sleep and/or alertness, or were diagnosed with an immunodeficiency syndrome or any auto‐immune or auto‐inflammatory disease except for atopic disease (Lammers‐van der Holst et al., 2022).

Out of the 165 volunteers who were screened for eligibility, 116 were excluded and 49 were included in the study (Figure S1). The initial goal was to enrol a total of 100 participants in the study (50 day workers and 50 night shift workers) (Lammers‐van der Holst et al., 2022). At the time this study was developed, no data were available to predict the difference in the COVID‐19 vaccine‐induced immune response between the two groups. Therefore, no exact power calculation could be provided for this study cohort. We estimated the sample size based on the results of a previous study by Lange et al. (2003), where a total of 19 participants were included. The Netherlands, like many other countries, had placed their healthcare workers high on the priority list for vaccine access (Thorsteinsdottir & Madsen, 2021). As the recruitment of participants for this study occurred at the onset of the national scaled COVID‐19 vaccination campaign in the Netherlands, the majority of our target group, consisting of healthcare workers and first responders, interested in participating were already vaccinated. As a result, we were not able to recruit the desired 50 participants per group (day workers/night shift workers). We excluded four participants for further analysis due to high spike (S)‐specific antibody levels at baseline (*n* = 2), indicative of a recent SARS‐CoV‐2 infection, or because in hindsight, they did not work night shifts directly after vaccination (night shift workers, *n* = 2). In the end, 24 day workers and 21 night shift workers were included in the analysis. All included participants were COVID‐19 naïve and had not yet received a COVID‐19 vaccination.

### 2.3 Study design

**FIGURE 1:**
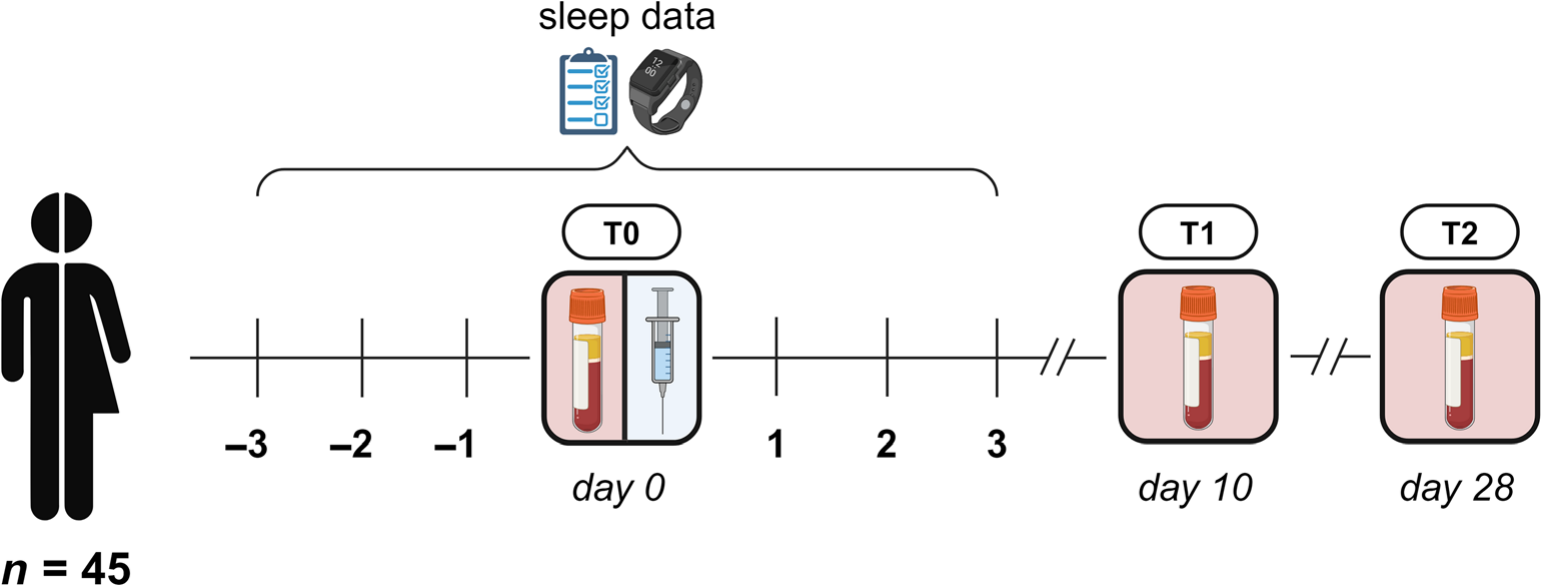
Schematic overview of the study protocol. Participants were vaccinated intramuscularly with the messenger RNA (mRNA)‐1273 vaccine. Blood samples were collected at baseline, and ±10 (T1) and ±28 days (T2) after vaccination. Sleep‐wakefulness was measured for a period of 7 days, starting 3 days prior to vaccination, using actigraphy and daily electronic diaries.

At baseline, participants completed a questionnaire in Castor Electronic Data Capture (Castor, 2019) including demographics and chronotype. To assess whether someone was a morning, an evening or an intermediate type, a validated single question regarding self‐reported chronotype was used (Roenneberg et al., 2003). Participants were vaccinated intramuscularly with the mRNA‐1273 vaccine between April and June 2021. The time‐of‐day that day workers received their vaccination ranged from 8:00 a.m. to 9:00 p.m. (median 11:00 a.m.). In the case of night shift workers, timing of vaccination varied from 10:00 a.m. to 6:00 p.m. (median 2:00 p.m.), where the majority received their vaccination in the afternoon. The vaccination for night shift workers was scheduled during the day, just before they began working at least two consecutive night shifts, starting that evening. We chose to vaccinate the night shift workers prior to rather than directly after their night shifts to be able to vaccinate both groups during day‐time in order to reach comparable vaccination times. This design also ensured that the period of circadian misalignment due to night shift work occurred during the early development of the vaccine‐induced immune response. Moreover, vaccinating the night shift workers after their night shifts would be practically challenging, as this would interfere with their recovery sleep and create an extra bias in the analysis. Blood samples were collected at baseline (T0; 1–8 days prior to vaccination), and ±10 (T1) and ±28 days (T2) after vaccination, to determine the primary immune response to vaccination (Figure 1). We focused on the immune response elicited by one injection of the mRNA‐1273 vaccine. Although it could be argued that the first dose of the vaccine induces a suboptimal immune response with high variability, it reflects the early stages of immune activation and is an essential component of the overall immune effect (Grunau et al., 2022). In contrast, the immune response following the second dose typically stabilises and plateaus, with more consistent immune activation across individuals (Grunau et al., 2022). However, this also limits the ability to detect minor differences between groups as the immune system has already been strongly primed and smaller effects would be potentially overshadowed by the amplified immune stimulation (Almeida et al., 2024; Grunau et al., 2022). Participants were asked to wear an activity monitor for a period of 7 days, starting 3 days prior to their vaccination. At the same time, participants received a daily electronic diary through Castor Electronic Data Capture to report work times, sleep duration, and sleep quality. For a more detailed description of the study design, see the previously published protocol (Lammers‐van der Holst et al., 2022).

### 2.4 Sleep measurements

Participants wore an activity monitor (Motionwatch 8; *n* = 26 or Philips Actiwatch Spectrum PRO; *n* = 19) on their non‐dominant wrist for a period of 7 days, starting 3 days prior to their vaccination. During this time, rest and activity levels were recorded in 60‐s epochs. Total sleep time (TST) per 24 h was analysed using MotionWare (version 1.1.20, CamNTech Ltd., Cambridge, UK) or Actiware (version 1.3.17, Philips, Eindhoven, The Netherlands) software, with manual editing of sleep episodes. When a participant had multiple sleep episodes on 1 day (*n* = 17), the episodes >30 min were combined to determine TST. In addition, the daily electronic diary recorded bed‐in and bed‐out times, as well as the number and duration of naps. The sleep data from the electronic diaries was used to confirm the objective sleep duration and fill in missing data points. In all, 86.4% of the main sleep data originated from the actiwatches and 11.4% from the electronic diaries. Of the data on the remaining sleep episodes, such as naps, 70.4% was recorded using actigraphy and 29.6% originated from the electronic diaries. For four participants and a total of 7 days (2.2% of the total main sleep data), no actigraphy or diary entry data were present. Here, TST averaged over the remaining timepoints was used to complete the data. The daily electronic diary was also used to document subjective sleep quality, which was measured with a 5‐point Likert scale ranging from 1 (‘very good’) to 5 (‘very poor’).

### 2.5 Immunogenicity

The S1‐specific IgG levels were measured using the LIAISON SARS‐CoV‐2 TrimericS IgG assay (Diasorin, Italy). The lower limit of detection (LLoD) was set at 4.81 binding antibody units (BAU)/mL and the cut‐off for positive IgG levels was set at 33.8 BAU/mL, according to manufacturer's instructions.

The SARS‐CoV‐2‐specific interferon‐γ (IFN‐γ) production by T cells was assessed using the commercially available IFN‐γ release assay QuantiFERON test (QIAGEN, Hilden, Germany), as previously described (Sanders et al., 2022; Tan et al., 2023). In short, 1 mL heparinised whole blood obtained at each timepoint was incubated at 37°C for 20–24 h in tubes containing peptides representing the whole S‐protein to induce CD4^+^ and CD8^+^ T‐cell responses. A mitogen positive control and negative control (vehicle, Nil) were included for each sample. Following incubation, plasma was obtained and IFN‐γ levels were determined using the QuantiFERON anti‐IFN‐γ enzyme‐linked immunosorbent assay (ELISA) kit (QIAGEN, Hilden, Germany). The LLoD was set at 0.01 international units (IU)/mL and the responder cut‐off was set at 0.15 IU/mL, according to manufacturer's instructions.

### 2.6 Statistical analysis

Normality was assessed by the Shapiro–Wilk test. Differences in baseline characteristics between day workers and night shift workers were assessed with the Wilcoxon rank‐sum test and the Fisher's exact test for respectively continuous and categorical variables. The *t*‐test was performed to establish differences in sleep duration and sleep quality between day workers and night shift workers, as we found these variables to follow a normal distribution. The Wilcoxon rank‐sum test was used to assess difference in S‐specific immune responses between the two groups. The effect of blood collection time on the S‐specific immune responses was determined using the Kruskal–Wallis test, followed by a post hoc Dunn test with the Bonferroni correction for multiple testing. The association between sleep duration and sleep quality, and the immune response 10 and 28 days following vaccination was assessed by multivariate linear regression models including β‐coefficients and 95% confidence intervals (CIs). We estimated a main effect model for the association between sleep and the immune response, for day workers and night shift workers. Furthermore, another model including an interaction effect was estimated to analyse the association between sleep, day worker or night shift worker dummy, and the immune response. All statistical analyses were performed using RStudio software (version 4.0.5). A *p* < 0.05 was considered statistically significant. BioRender and Graphpad Prism software (version 9.5.1) were used to visually present the data.

## 3 RESULTS

### 3.1 Characteristics of the study population

Characteristics of the study population are summarised in Table 1. There were no significant differences in sex, age, and body mass index between day workers and night shift workers. However, there was a significant effect of group type (day workers/night shift workers) on chronotype (*p* = 0.016). Night shift workers mainly classified themselves as (moderate) evening types, whereas day workers were more evenly distributed over the different chronotypes. The median vaccination time was 11:00 a.m. for day workers and 2:00 p.m. for night shift workers. All night shift workers had at least 2 years of experience in working night shifts. Out of the 21 night shift workers, six worked three consecutive night shifts after their vaccination, 14 worked two consecutive night shifts and one worked only one night shift. As the analyses with and without the data from the participant who worked only one night shift yielded similar results, we included their data in the final analysis.

**TABLE 1:** Characteristics, baseline immune response and sleep data of the study population.

Variable, % or median (IQR)	Total (*n* = 45)	Day workers (*n* = 24)	Night shift workers (*n* = 21)
Sex			
Male	44.4	41.7	47.6
Female	55.6	58.3	52.4
Age, years	32.6 (27.6–41.6)	37.1 (28.6–44.6)	32.6 (25.6–37.6)
BMI, kg/m^2^	23.0 (21.8–25.5)	23.0 (22.3–25.6)	22.7 (21.3–25.5)
Chronotype^a^			
Morning active	22.2	33.3	9.5
Moderate morning active	11.1	20.8	0.0
Neutral	22.2	16.7	28.6
Moderate evening active	17.8	16.7	19.0
Evening active	26.7	12.5	42.9
Immunology			
Baseline antibody levels, BAU/mL	4.81 (4.81–4.81)	4.81 (4.81–4.81)	4.81 (4.81–4.81)
Baseline IFN‐γ levels, IU/mL	0.06 (0.04–0.13)	0.06 (0.05–0.09)	0.06 (0.02–0.13)
TST, min^b^	418 (389–436)	426 (414–442)^a^	393 (370–425)^a^
Diary‐based sleep quality^b^	2.57 (2.29–2.86)	2.51 (2.29–2.89)	2.57 (2.20–2.86)

Abbreviations: BAU, binding antibody units; BMI, body mass index; IFN‐γ, interferon‐γ; IQR, interquartile range; IU, international units; TST, total sleep time.

^a^ Statistically significant difference (*p* < 0.05) between day workers and night shift workers tested using the Fisher's exact test (chronotype) or the *t*‐test (TST).

^b^ Values averaged over 7 days of data collection.

### 3.2 Sleep outcomes

**FIGURE 2:**
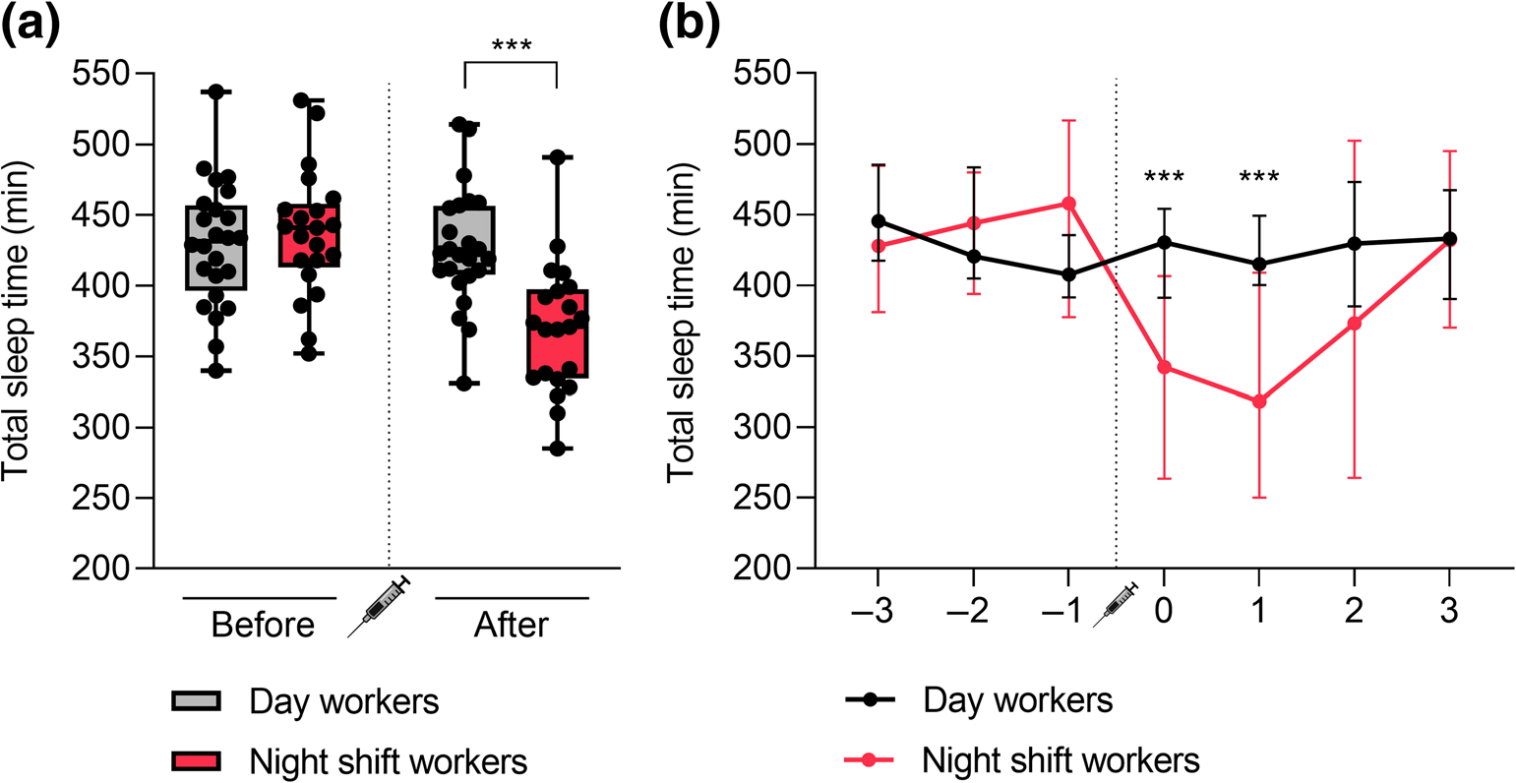
Total sleep time (TST) of day workers and night shift workers around vaccination day. (a) TST of day workers and night shift workers before and after vaccination. Boxplots are presented as the median TST ± maximum/minimum values. Every dot represents the average TST over either the 3 days before or the 4 days after vaccination for one individual. (b) TST of day workers and night shift workers measured over a period of 7 days around the moment of vaccination. Values are presented as the median TST ± interquartile range. ***p < 0.001 (*t*‐test).

Prior to vaccination, both day workers and night shift workers stuck to night‐time sleeping. Night shift workers shifted to daytime sleeping on the days they worked night shifts, which were scheduled immediately following vaccination. The median TST, calculated as the median of the average TST values over 7 days of measurement for every individual, over 7 days around vaccination was significantly lower in night shift workers (393 min) versus day workers (426 min) (*p* = 0.018) (Table 1). Next, we analysed the median sleep duration of day workers and night shift workers before and after vaccination separately. While there was no significant difference in the median TST over the 3 days before vaccination between the two groups (431 min for day workers versus 441 min for night shift workers, *p* = 0.530), night shift workers had a significantly lower median TST over the 4 days after vaccination (422 min for day workers versus 371 min for night shift workers, *p* < 0.001) (Figure 2a). We also assessed the difference in median sleep duration between day workers and night shift workers on each separate day of measurement (Figure 2b). On the 2 days directly after vaccination, the median TST was significantly lower in night shift workers (342 and 318 min) than day workers (431 and 415 min) (both *p* < 0.001). On the remaining days, no significant difference in median TST between the two groups was found. We also did not find a significant difference in median sleep quality over 7 days around vaccination between day workers (2.51) and night shift workers (2.57) (*p* = 0.844) (Table 1).

### 3.3 The SARS‐CoV‐2‐specific humoral and cellular immune responses

**FIGURE 3:**
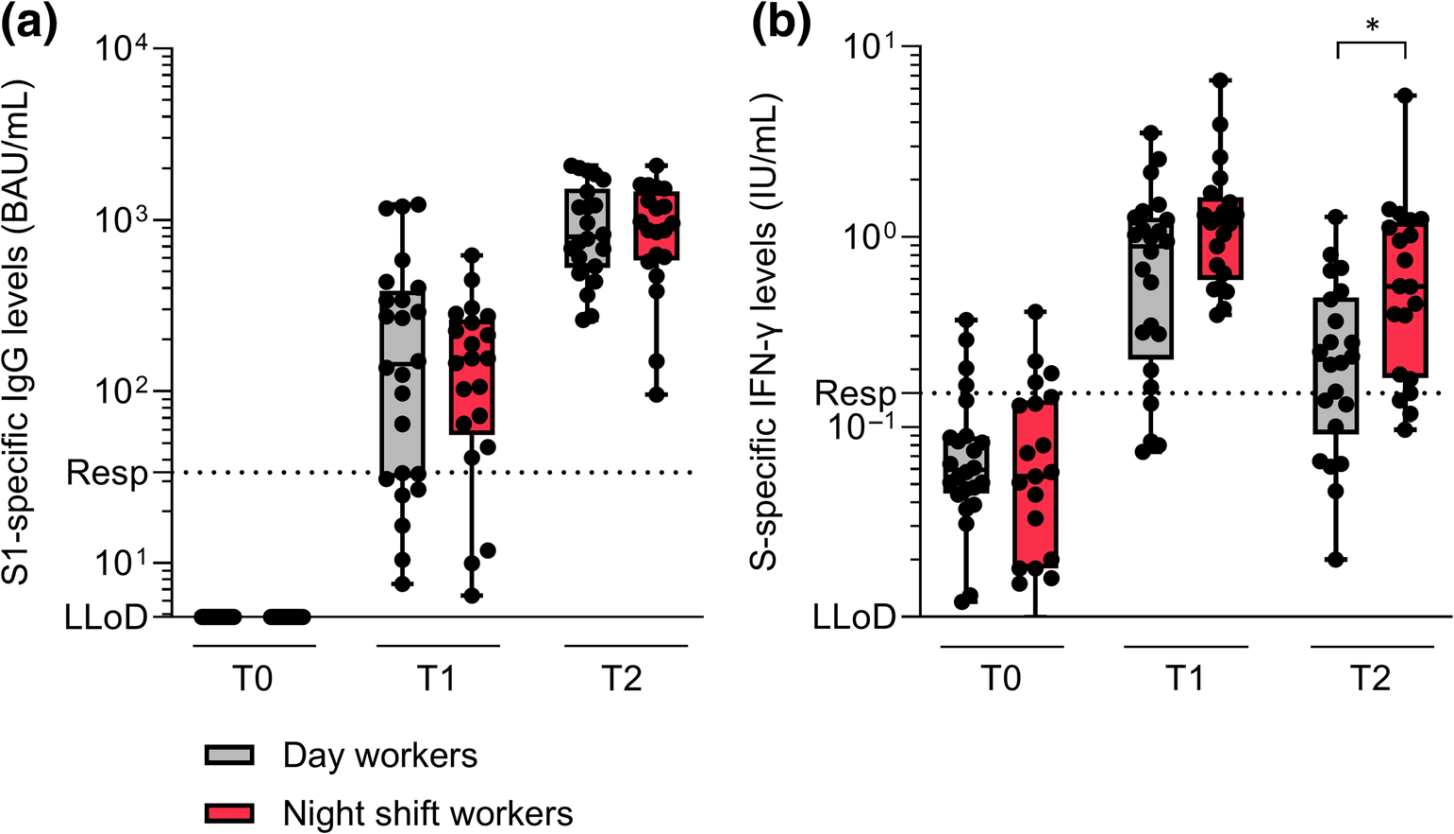
Spike (S)‐specific antibody (a) and T‐cell (b) responses in day workers and night shift workers ±10 (T1) and ±28 days (T2) after vaccination. A. S1‐specific immunoglobulin G (IgG) antibody levels (binding antibody units (BAU)/mL) in day workers and night shift workers at baseline (T0), and ±10 (T1) and ±28 days (T2) after immunisation. Boxplots are presented as the median TST ± maximum/minimum values. Every dot represents one individual. The lower limit of detection (LLoD) was set at 4.81 BAU/mL and the cut‐off for positive IgG levels (Resp) was set at 33.8 BAU/mL. B. S‐specific interferon‐γ (IFN‐γ) production by T cells (international units (IU)/mL) in day workers and night shift workers at (T0), and ±10 (T1) and ±28 days (T2) after immunisation. Boxplots are presented as the median TST ± maximum/minimum values. Every dot represents one individual. The LLoD was set at 0.01 IU/mL and the responder cut‐off (Resp) was set at 0.15 IU/mL. *p < 0.05 (Wilcoxon rank‐sum test).

The S1‐specific antibody and T‐cell responses in day workers and night shift workers were measured following the first injection of the mRNA‐1273 vaccine. None of the participants in either the day or night group had pre‐existing S1‐specific antibody levels at baseline (Table 1, Figure 3a). At 10 days after immunisation, S1‐specific IgG binding antibodies were detectable in both day workers (median [interquartile range, IQR] 143.5 [32.5–356.3] BAU/mL) and night shift workers (median [IQR] 155.0 [64.4–251.0] BAU/mL). Antibody titres further increased in both groups at the third timepoint, 28 days following vaccination (median [IQR] 799.0 [553.8–1400.0] BAU/mL in day workers versus 914.5 [599.5–1357.5] BAU/mL in night shift workers) (Figure 3a). No significant difference in antibody levels was found between day workers and night shift workers at each timepoint.

No baseline T‐cell responses were observed in either day workers or night shift workers before vaccination (Table 1, Figure 3b). S‐specific IFN‐γ production by T cells peaked 10 days after immunisation in both day workers (median [IQR] 0.89 [0.28–1.24] IU/mL) and night shift workers (median [IQR] 1.19 [0.64–1.52] IU/mL). At this timepoint, the T‐cell response tended to be higher in night shift workers versus day workers (*p* = 0.057). Although the S‐specific IFN‐γ levels decreased in both groups 28 days following vaccination (median [IQR] 0.23 [0.11–0.44] IU/mL in day workers versus 0.55 [0.19–1.14] IU/mL in night shift workers), night shift workers had significantly higher IFN‐γ levels in comparison to day workers (*p* = 0.013) (Figure 3b).

There was variability in the timing of blood collection amongst the participants. Blood collection at T1 and T2 ranged from respectively 8–14 and 22–35 days after vaccination. No significant difference was found in the timing of blood collection between day workers and night shift workers, both at T1 (*p* = 0.931) and T2 (*p* = 0.563). The timing of blood collection only had an effect on the antibody response at T1 (Figure S2). There was a significant difference in S1‐specific antibody levels between individuals sampled after 8 and 14 days (*p* = 0.009), and individuals sampled after 9 and 14 days (*p* < 0.001).

### 3.4 Association between sleep and SARS‐CoV‐2‐specific immune responses

**FIGURE 4:**
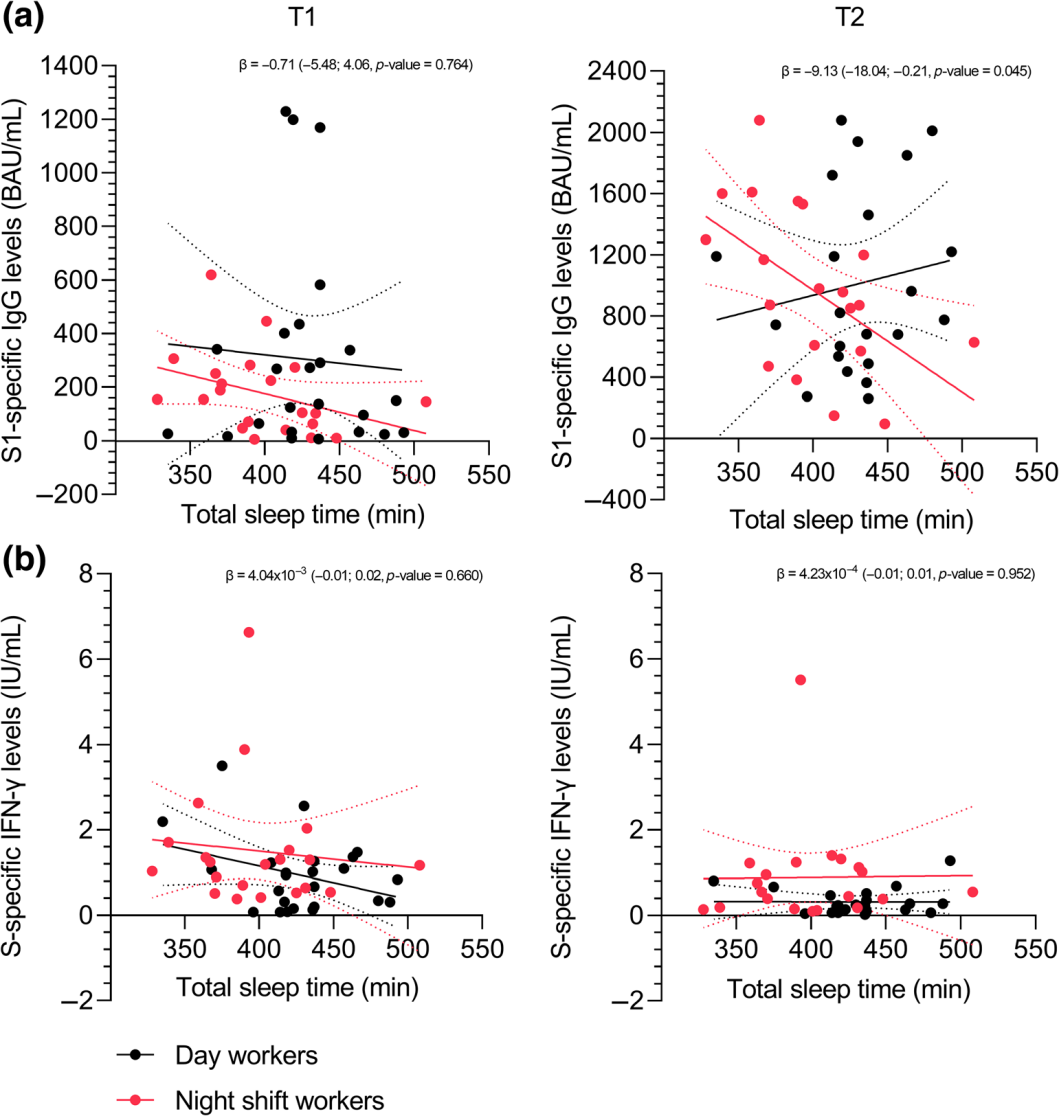
Association between total sleep time (TST) and severe acute respiratory syndrome coronavirus‐2 (SARS‐CoV‐2)‐specific immune responses. (a) Simple linear regression model of the relationship between TST and Spike (S1)‐specific immunoglobulin G (IgG) antibody levels (binding antibody units (BAU)/mL) at T1 (left) and T2 (right), for day workers and night shift workers. (b) Simple linear regression model of the relationship between TST and S‐specific interferon‐γ (IFN‐γ) production by T cells (international units (IU)/mL) at T1 (left) and T2 (right), for day workers and night shift workers. The continuous lines represent the linear regression models. The dashed lines represent the 95% confidence intervals.

The associations between sleep duration (TST) and SARS‐CoV‐2‐specific immune responses are visualised in Figure 4. The multivariate linear regression model showed no significant association between TST and S1‐specific IgG levels 10 days after vaccination (β = −1.02, 95% CI −3.38 to 1.33; *p* = 0.385). Also no significant association was found between group type (day worker/night shift worker dummy variable) and the antibody response 10 days after vaccination (β = −155.99, 95% CI −348.46 to 36.48; *p* = 0.109) (Figure 4a, Table S1). There was no significant interaction effect between TST and group type (day worker/night shift worker dummy variable) for S1‐specific IgG levels 10 days after vaccination (β = −0.71, 95% CI −5.48 to 4.06; *p* = 0.764) (Figure 4a, Table S2).

The multivariate linear regression model showed no significant association between TST and S‐specific IFN‐γ levels 10 days after vaccination (β = −0.01, 95% CI −0.01 to 0.00; *p* = 0.213). Also no significant association was found between group type (day worker/night shift worker dummy variable) and S‐specific IFN‐γ levels 10 days after vaccination (β = 0.40, 95% CI −0.34 to 1.15; *p* = 0.279) (Figure 4b, Table S1). There was no significant interaction effect between TST and group type (day worker/night shift worker dummy variable) for S‐specific IFN‐γ 10 days after vaccination (β = 4.04 × 10^−3^, 95% CI −0.01 to 0.02; *p* = 0.660) (Figure 4b, Table S2).

The multivariate linear regression models for antibody levels and T‐cell responses 28 days after vaccination showed similar results to the 10 days post‐vaccination models and are presented in Figure 4 and Tables S1 and S2. Similar results were obtained when TST over the 4 days after vaccination was used instead of the whole 7‐day period (data not presented here). No significant associations were found between sleep quality and SARS‐CoV‐2 specific immune responses (Figure S3, Tables S1 and S2).

## 4 DISCUSSION

Our findings show that day workers and night shift workers induce similar SARS‐CoV‐2‐specific antibody responses following the first injection of the mRNA‐1273 vaccine, despite significant differences in sleep duration directly after vaccination. Virus‐specific T‐cell responses were significantly higher in night shift workers than day workers 28 days after immunisation.

It is well known that night shift workers report more sleep disturbances than day workers (Akerstedt, 2003; Akerstedt & Wright Jr., 2009). A previous study by Brum et al. (2020) found a significant difference in sleep duration between day and night shift workers. Furthermore, two independent studies reported that night shift workers sleep less after a night shift than after day work or on days off (Athanasiou et al., 2023; Garde et al., 2020). In line with this, we found that the median TST was significantly lower in night shift workers (342 and 318 min) than day workers (431 and 415 min) on the 2 days immediately after vaccination, which coincided with the days that night shift workers worked night shifts. No difference in sleep duration between the two groups was found on the remaining days of measurement.

We found that day workers and night shift workers had similar S1‐specific IgG antibody levels at 10 and 28 days after immunisation. SARS‐CoV‐2‐specific IFN‐γ production by T cells tended to be higher in night shift workers than day workers, but the difference was only significant 28 days after vaccination. Whether both groups were also equally well protected against a SARS‐CoV‐2 breakthrough infection during this 28‐day period was not determined. Even though beyond the scope of this paper, it would be interesting to closely examine the cellular phenotype and functionality of day workers versus night shift workers to better understand the underlying factors driving the differences in T‐cell responses. As previous research has established that night shift workers have more CD8^+^ T cells than day workers (Loef, Nanlohy, et al., 2019), future studies focusing on potential differences in functionality within this T‐cell subset (naïve/central memory/effector memory T cells) and cytokine production would give valuable insights. Contrary to our results, Ruiz et al. (2020) showed that night workers had a weaker humoral immune response than day workers 28 and 56 days following vaccination with the meningococcal group C conjugate vaccine. This discrepancy with our study could be explained by the different vaccines used. It is also important to point out that the night shift workers in our study, but also the study of Ruiz et al. (2020), were vaccinated following a period of rest and thus not in a state of acute circadian misalignment. A recent study found no association between working night shifts on the 2 days before vaccination and the SARS‐CoV‐2‐specific antibody response (Athanasiou et al., 2023), although the number of night shift workers who worked night shifts before their vaccination was small. Together, these findings indicate that working night shifts directly before or after vaccination has no detrimental acute effects on the immune response to vaccination. To gain more understanding on the effects of long‐term shift work on the immune response to vaccination, it would be interesting to compare shift workers with different histories of night shift work in future studies.

Vaccination responses in humans seem to be under circadian control (Wang, Lutes, et al., 2022). People immunised against COVID‐19 in the morning and afternoon had less breakthrough infections than those vaccinated in the evening (Hazan et al., 2023). However, no antibody levels or T‐cell responses were measured in this large Israelian cohort. Although not statistically significant, timing of vaccination differed between the day workers and night shift workers in our study. Where 18 out of 24 day workers were vaccinated before 2:00 p.m., this was the case for only nine out of 21 night shift workers. A COVID‐19 vaccination study in which healthcare workers were vaccinated with either the Pfizer mRNA or AstraZeneca adenovirus vaccine found that anti‐S antibody responses were higher in people who received their injection later in the day (Wang, Balfe, et al., 2022). This might explain why we did not observe large differences in immune responses between the two groups.

There is growing evidence that sleep exerts a strong regulatory influence on the immune system. Sleep is thought to stimulate interleukin 12 production by antigen‐presenting cells, thereby strengthening the interaction between these antigen‐presenting cells and T cells, resulting in stronger T‐cell responses (Besedovsky et al., 2012). Besides, sleep seems to boost immunological memory (Lange et al., 2011). In the present study, we did not find an association between sleep duration or sleep quality and primary immunogenicity of the mRNA‐1273 vaccine. These results are in line with a recent study by Athanasiou et al. (2023), where no association between sleep duration or sleep quality and SARS‐CoV‐2‐specific antibody levels was observed, after adjusting for confounders. In contrast, a meta‐analysis, which included seven different studies on either hepatitis or influenza vaccination, found a significant association between short sleep duration and reduced antibody levels after vaccination. However, it is important to point out that the participants in the experimental studies were either completely sleep deprived the night before or after vaccination or were restricted to 4 h sleep/night at the time of vaccination. For the analysis on self‐reported short sleep duration, a maximum of 6 h of sleep/night was taken. The level of sleep loss that the night shift workers in our study experienced might have been too little to see an effect on the SARS‐CoV‐2‐specific immune response. Moreover, we specifically focused on the primary immune response to vaccination. Lange et al. (2011) observed that sleep deprivation on the night following hepatitis A vaccination impaired the antigen‐specific humoral and cellular immune responses 8 weeks after immunisation, but not earlier. The beneficial effect of sleep on the vaccine‐induced immune response became even more prominent after booster vaccinations. Collectively, this might indicate that sleep loss directly after vaccination has a less profound effect on the primary immune response than on the long‐term immune response.

This study has a few limitations. First, the recruitment of participants occurred at the onset of the large‐scale COVID‐19 vaccination campaign in the Netherlands. As a result, a lot of people interested in participating in the study were already vaccinated, and the sample size turned out to be smaller than our desired 50 participants/group (day workers/night shift workers). This also limited the possibility to correct for factors such as chronotype and time‐of‐day of vaccination, which differed considerably between the day and night workers in this study. To draw more definite conclusions on the effect of sleep and shift work on a primary immune response to vaccination, a larger sample size is required. Second, the high workload of healthcare workers and police officers during the peak of the COVID‐19 pandemic made it difficult to plan the blood draw moments exactly at 10 and 28 days after vaccination. As result, there was variability in the day of blood sampling following vaccination amongst the participants. However, because no significant difference was observed in the timing of blood collection between day workers and night shift workers, we believe these findings do not affect the conclusions of this study. Finally, we want to emphasise that the increased S‐specific IFN‐γ levels in night shift workers as compared to day workers should be interpreted with caution. The cellular immune response elicited by the mRNA‐1273 vaccine was measured using the IGRA QuantiFERON test, which does not correct for the number of cells. Loef, Nanlohy, et al. (2019) found that night shift workers who recently worked night shifts have significantly more T cells and CD8^+^ T cells/μL blood than non‐shift workers. It could therefore be possible that the higher IFN‐γ levels in night shift workers is just a reflection of more T cells being present in the blood. More information on the timeline of night shifts around the moments of blood collection would be needed to support this hypothesis.

In summary, day workers and night shift workers had similar SARS‐CoV‐2‐specific antibody responses following the first injection of the mRNA‐1273 vaccine, despite significant differences in sleep duration immediately after vaccination. Virus‐specific T‐cell responses were significantly higher in night shift workers than day workers at 28 days after immunisation. There was no difference in sleep quality between the two groups. No association between sleep duration, sleep quality and SARS‐CoV‐2‐specific immune responses was found. These findings indicate that shift work‐induced sleep loss and night shift work have little to no effect on the primary immune response to mRNA‐based COVID‐19 vaccination.

## AUTHOR CONTRIBUTIONS

**Tamara M. J. Brouwers:** Formal analysis; writing – original draft; writing – review and editing; visualization. **Ümmü Gülsüm Çobanoğlu:** Formal analysis; investigation; writing – original draft; writing – review and editing; visualization. **Daryl Geers:** Formal analysis; visualization; writing – review and editing. **Wim J. R. Rietdijk:** Methodology; writing – review and editing; visualization. **Lennert Gommers:** Investigation; writing – review and editing. **Susanne Bogers:** Investigation; writing – review and editing. **Gert Jan Lammers:** Conceptualization; writing – review and editing. **Gijsbertus T. J. van der Horst:** Conceptualization; writing – review and editing. **Inês Chaves:** Conceptualization; writing – review and editing. **Corine H. GeurtsvanKessel:** Conceptualization; writing – review and editing. **Birgit C. P. Koch:** Conceptualization; writing – review and editing. **Rory D. de Vries:** Methodology; writing – review and editing. **Debbie van Baarle:** Writing – review and editing. **Hugo M. van der Kuy:** Conceptualization; writing – review and editing; supervision; investigation. **Heidi M. Lammers‐van der Holst:** Conceptualization; methodology; investigation; writing – review and editing; supervision.

## FUNDING INFORMATION

This publication is part of the project BioClock (with project number 1292.19.077) of the research programme Dutch Research Agenda: Onderzoek op Routes door Consortia (NWA‐ORC), which is (partly) financed by the Dutch Research Council (NWO).

## CONFLICT OF INTEREST STATEMENT

The authors have no conflicts of interests to declare.

## Supporting information


**Data S1.** Supporting Information.

## Data Availability

The data that support the findings of this study are available from the corresponding author upon reasonable request.
