# Supplementary material for: The effect of sleep and shift work on the primary immune response to messenger RNA‐based COVID‐19 vaccination
Source: J Sleep Res. 2024 Dec 10;34(4):e14431. doi: 10.1111/jsr.14431 (PMC12215213; doi:10.1111/jsr.14431)
Supplement: Supplementary file 1 — Data S1. Supporting Information. [file JSR-34-e14431-s001.docx]

**Supplementary Material**

**
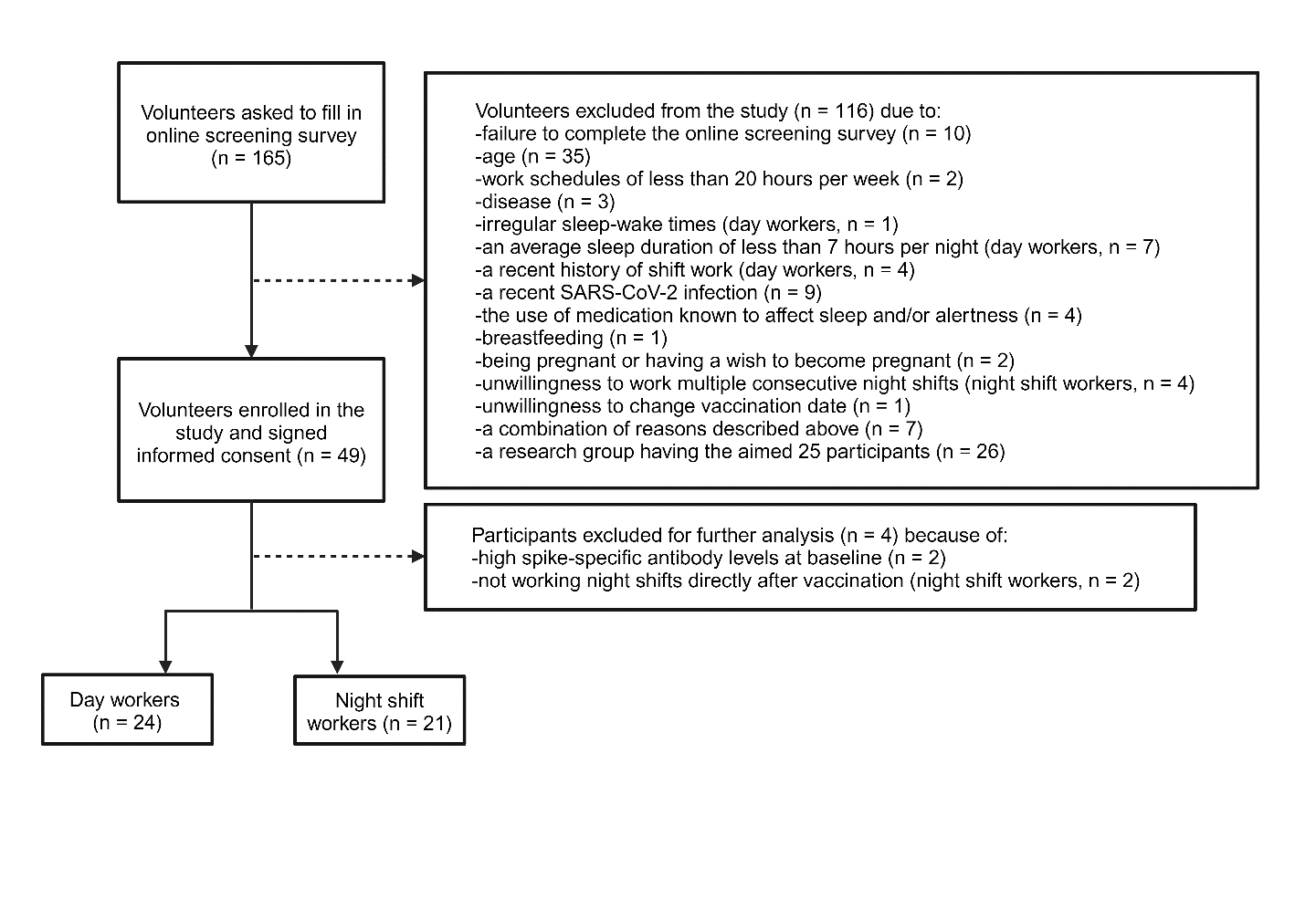
Supplementary figures**

**Figure S1.** Flow chart of the study population. Out of the 165 volunteers who were screened for eligibility, 116 were excluded and 49 were included in the study. Four participants were later excluded for further analysis due to high spike-specific antibody levels at baseline (n = 2), indicative of a recent SARS-CoV-2 infection, or because in hindsight, they did not work night shifts directly after vaccination (night shift workers, n = 2). In the end, 24 day workers and 21 night shift workers were included in the analysis.

**
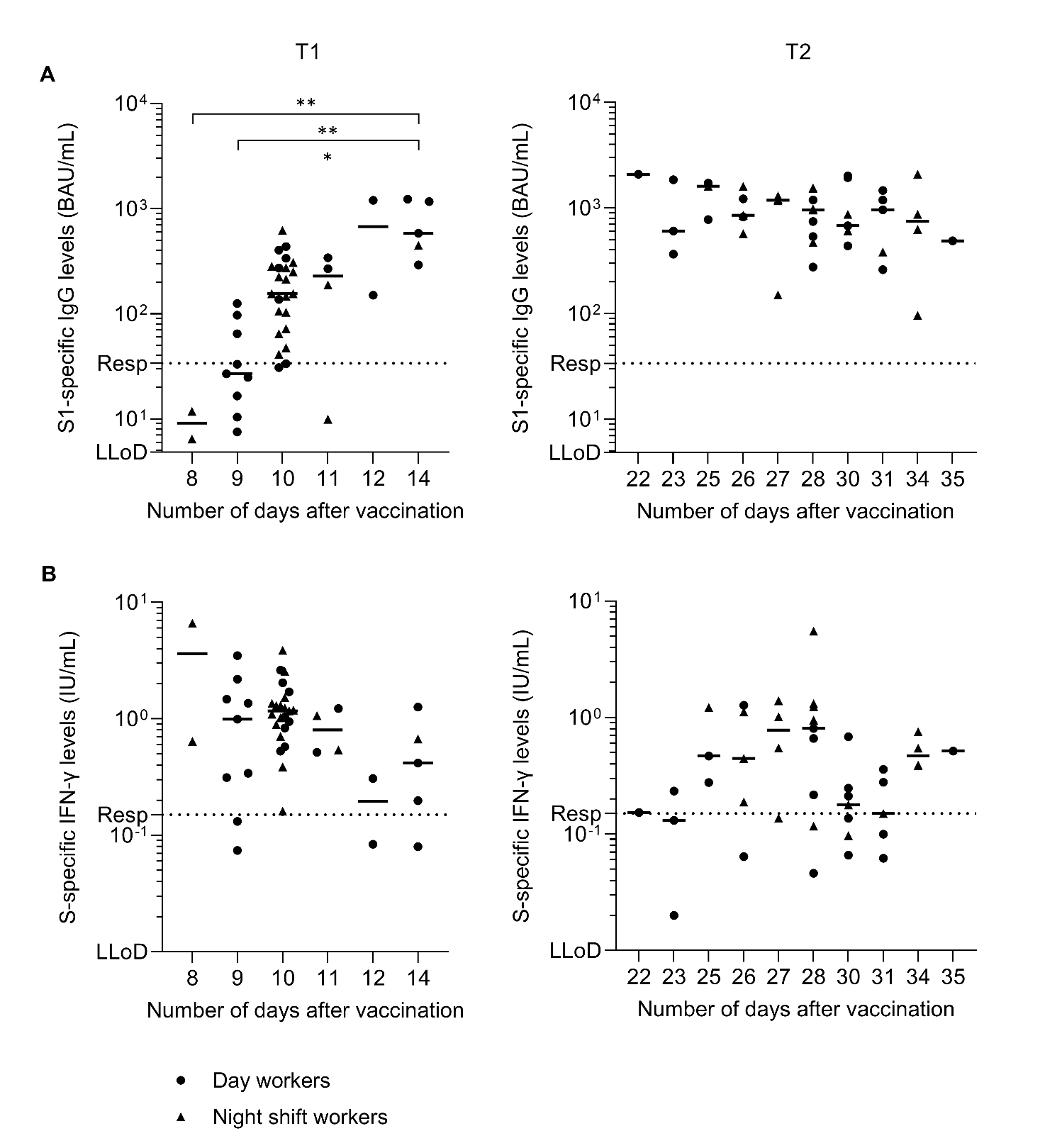

Figure S2.** Effect of blood collection time on the spike (S)-specific antibody (A) and T cell responses (B). A. The effect of blood collection time at T1 (left) and T2 (right) on the S1-specific IgG antibody levels (BAU/mL). Every dot represents one individual. The lower limit of detection (LLoD) was set at 4.81 BAU/mL and the cut-off for positive IgG levels (Resp) was set at 33.8 BAU/mL. B. The effect of blood collection time at T1 (left) and T2 (right) on the S-specific IFN-γ production by T cells (IU/mL). Every dot represents one individual. The LLoD was set at 0.01 IU/mL and the responder cut-off (Resp) was set at 0.15 IU/mL. **p < 0.01 (Kruskal-Wallis test, followed by a post-hoc Dunn test with Bonferroni correction for multiple testing).

**
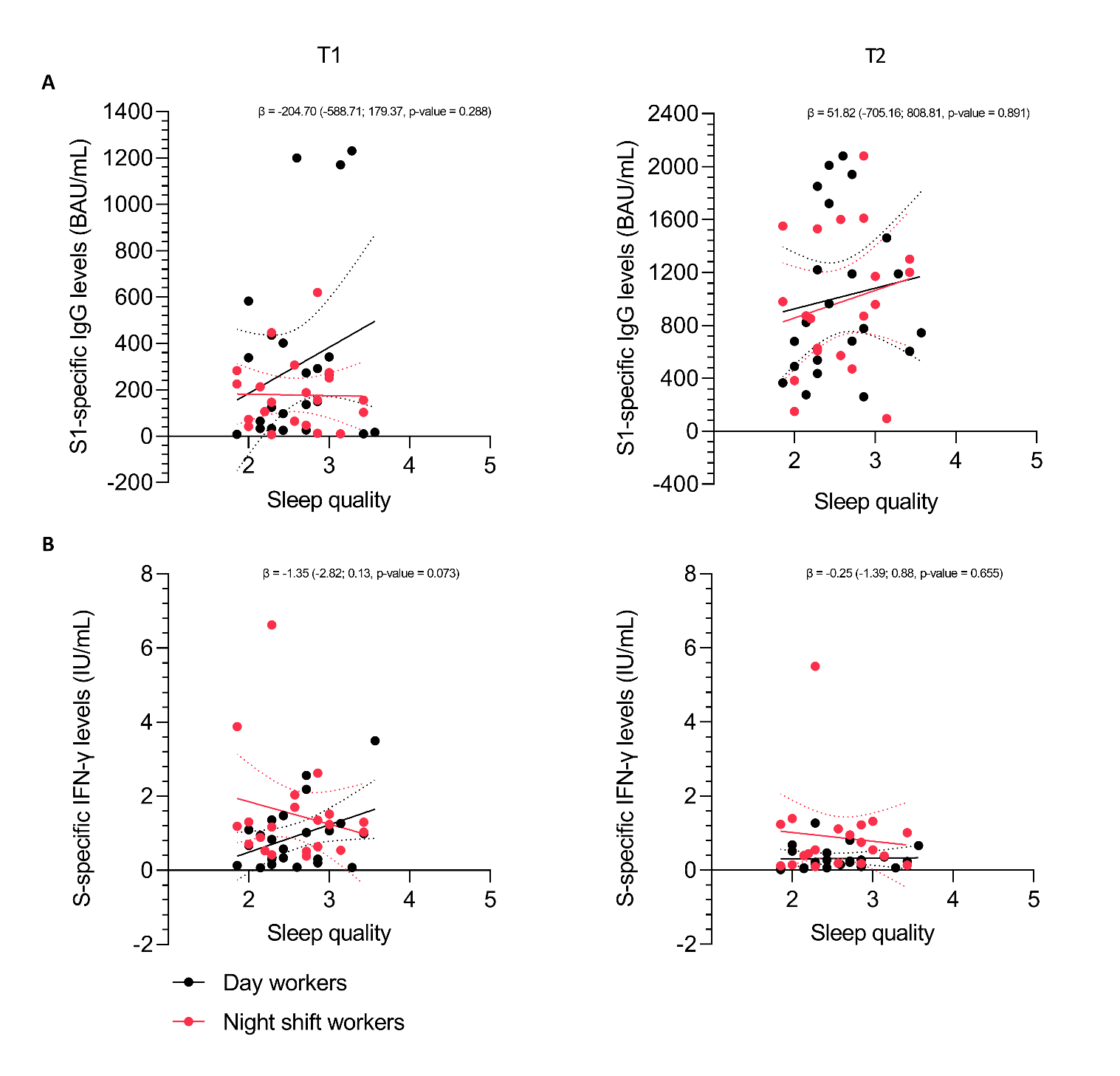
**

**Figure S3.** Association between sleep quality and SARS-CoV-2 specific immune responses. A. Simple linear regression model of the relationship between sleep quality and spike (S1)-specific IgG antibody levels at T1 (left) and T2 (right), for day workers and night shift workers. B. Simple linear regression model of the relationship between sleep quality and S-specific IFN-γ production by T cells levels at T1 (left) and T2 (right), for day workers and night shift workers. The continuous lines represent the linear regression models. The dashed lines represent the 95% confidence intervals.

**Supplementary tables**

**Table S1**

Main effect model of the association between sleep duration (*total sleep time;* *TST*) and quality, and SARS-CoV-2-specific immune responses

Main effect model sleep duration = Immune response ~ TST + group type (day worker/night shift worker)

Main effect model sleep quality = Immune response ~ sleep quality + group type (day worker/night shift worker)

| **Variable** | **Antibody levels** | | **T cell response** | |
| --- | --- | --- | --- | --- |
|  | 10 days  n = 45 | 28 days  n = 42 | 10 days  n = 45 | 28 days  n = 42 |
| TST (min)^a^ | -1.02  (-3.38; 1.33) | -2.54  (-7.16; 2.08) | -0.01  (-0.01; 0.00) | 2.05x10^-4^  (-0.01; 0.01) |
| Group type  (day = reference) | -155.99  (-348.46; 36.48) | -120.50  (-502.33; 261.33) | 0.40  (-0.34; 1.15) | 0.57  (0.00; 1.15) |
| Adjusted R^2^ | 0.02 | -0.02 | 0.05 | 0.06 |
| F-statistic | F(2,42) = 1.39,  p-value = 0.261 | F(2,39) = 0.64,  p-value = 0.531 | F(2,42) = 2.17,  p-value = 0.127 | F(2,39) = 2.32,  p-value = 0.112 |
| Sleep quality^a^ | 100.01  (-92.10; 292.11) | 182.00  (-191.38; 555.39) | 0.09  (-0.67; 0.85) | -0.11  (-0.67; 0.45) |
| Group type  (day = reference) | -123.45  (-302.76; 55.85) | -39.56  (-392.92; 313.80) | 0.57  (-0.14; 1.28) | 0.57*  (0.03; 1.10) |
| Adjusted R^2^ | 0.02 | -0.02 | 0.02 | 0.06 |
| F-statistic | F(2,42) = 1.56,  p-value = 0.222 | F(2,39) = 0.51,  p-value = 0.604 | F(2,42) = 1.35,  p-value = 0.271 | F(2,39) = 2.40,  p-value = 0.104 |

TST total sleep time

min minutes

Values represent the estimates (95% confidence intervals) from the main effect model

^a^ TST averaged over seven days of data collection

* Statistically significant effect (p < 0.05)

**Table S2**

Interaction effect model of the association between sleep duration (*total sleep time;* *TST*) and quality, and SARS-CoV-2-specific immune responses

Interaction effect model sleep duration = Immune response ~ TST*group type (day worker/night shift worker)

Interaction effect model sleep quality = Immune response ~ sleep quality*group type (day worker/night shift worker)

| **Variable** | **Antibody levels** | | **T cell response** | |
| --- | --- | --- | --- | --- |
|  | 10 days  n = 45 | 28 days  n = 42 | 10 days  n = 45 | 28 days  n = 42 |
| TST (min)^a^ | -0.66  (-4.08; 2.76) | 2.46  (-4.14; 9.06) | -0.01  (-0.02; 0.01) | -2.68x10^-5^  (-0.01; 0.01) |
| Group type  (day = reference) | 138.83  (-1842.82; 2120.48) | 3683.54  (-50.37; 7417.46) | -1.27  (-8.91; 6.38) | 0.40  (-5.56; 6.35) |
| Interaction | -0.71  (-5.48; 4.06) | -9.13*  (-18.04; -0.21) | 4.04x10^-3^  (-0.01; 0.02) | 4.23x10^-4^  (-0.01; 0.01) |
| Adjusted R^2^ | 0.00 | 0.06 | 0.03 | 0.04 |
| F-statistic | F(3,41) = 0.94,  p-value = 0.432 | F(3,38) = 1.90,  p-value = 0.147 | F(3,41) = 1.48,  p-value = 0.234 | F(3,38) = 1.51,  p-value = 0.229 |
| Sleep quality^a^ | 198.50  (-67.90; 464.80) | 156.22 (-377.74; 690.17) | 0.74  (-0.29; 1.76) | 0.02  (-0.79; 0.82) |
| Group type  (day = reference) | 406.20  (-603.63; 1415.96) | -172.58  (-2148.25; 1803.09) | 4.05*  (0.17; 7.94) | 1.22  (-1.75; 4.18) |
| Interaction | -204.70  (-588.71; 179.37) | 51.82  (-705.16; 808.81) | -1.35  (-2.82; 0.13) | -0.25  (-1.39; 0.88) |
| Adjusted R^2^ | 0.03 | -0.05 | 0.07 | 0.04 |
| F-statistic | F(3,41) = 1.43,  p-value = 0.247 | F(3,38) = 0.34,  p-value = 0.797 | F(3,41) = 2.08,  p-value = 0.118 | F(3,38) = 1.64,  p-value = 0.197 |

TST total sleep time

min minutes

Values represent the estimates (95% confidence intervals) from the interaction effect model

^a^ TST averaged over seven days of data collection

* Statistically significant effect (p < 0.05)
